# Radiotherapy/Chemotherapy-Immunotherapy for Cancer Management: From Mechanisms to Clinical Implications

**DOI:** 10.1155/2023/7530794

**Published:** 2023-02-02

**Authors:** Qian Li, Xu Lei, Jing Zhu, Yanping Zhong, Junjie Yang, Jincheng Wang, Huabing Tan

**Affiliations:** ^1^Department of Urology, Huai'an Second People's Hospital and The Affiliated Huai'an Hospital of Xuzhou Medical University, Huai'an, China; ^2^Department of Infectious Diseases and Lab of Liver Disease, Renmin Hospital, Hubei University of Medicine, Shiyan, Hubei, China; ^3^Nanjing Drum Tower Hospital Clinical College of Nanjing Medical University, Nanjing, China; ^4^Renmin Hospital, Hubei University of Medicine, Shiyan, Hubei, China; ^5^Graduate School, Jinzhou Medical University, Jinzhou, Liaoning, China

## Abstract

Cancer immunotherapy has drawn much attention because it can restart the recognition and killing function of the immune system to normalize the antitumor immune response. However, the role of radiotherapy and chemotherapy in cancer treatment cannot be ignored. Due to cancer heterogeneity, combined therapy has become a new trend, and its efficacy has been confirmed in many studies. This review discussed the clinical implications and the underlying mechanisms of cancer immunotherapy in combination with radiotherapy or chemotherapy, offering an outline for clinicians as well as inspiration for future research.

## 1. Introduction

Normally, the immune system can recognize and clear tumor cells in the tumor microenvironment (TME), but tumor cells can suppress the human immune system to avoid being killed [1], which is an immune escape. To understand the complexity of tumor immunity, Chen and Mellman put forward the concept of the cancer-immunity cycle, which includes seven parts: (1) release of tumor antigen, (2) presentation of tumor antigen, (3) priming and activation of effector T cells, (4) migration of T cells to tumor tissues, (5) infiltration of T cells into tumor tissues, (6) recognition of tumor cells by T cells, and (7) removal of tumor cells [2]. Any abnormality in these procedures may bring about failed anti-tumor-immune circulation, thereby leading to immune escape. Tumors can promote immune tolerance, in which the recognition and killing of tumor cells by the immune system are inhibited.

Immunotherapy, including targeted antibodies, adoptive cell therapy, cancer vaccines, oncolytic viruses, and immunosuppressive agents, is a therapeutic method with which to restart the tumor-immunity cycle and restore the normal immune response to control and remove tumors. Due to its excellent efficacy, immunotherapy has received widespread attention. However, the proportion of people who respond to immunotherapy is low, and some patients even suffer from severe side effects.

**Figure 1:**
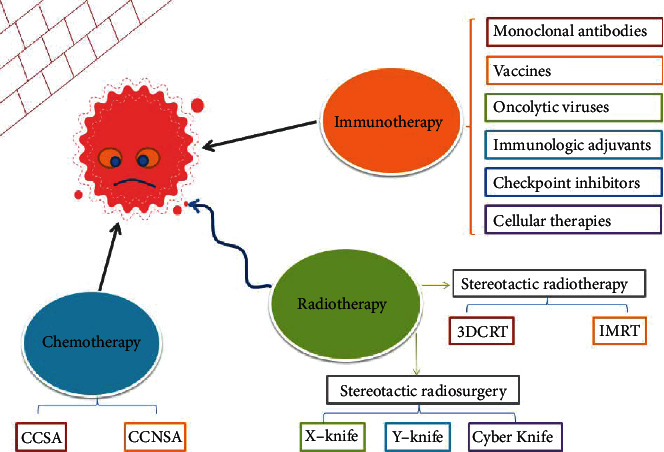
The triple strikes of cancer. Immunotherapy includes monoclonal antibodies, vaccines, oncolytic viruses, immunological adjuvants, checkpoint inhibitors, and cellular therapies. Stereotactic radiotherapy (SRT) and stereotactic radiosurgery (SRS) are the mainstream radiotherapy technologies. SRT includes three-dimensional conformal radiotherapy (3D CRT) and intensity-modulated radiotherapy (IMRT). SRS includes X-knife, Y-knife, and Cyber Knife. Chemotherapy includes cell cycle-specific agents (CCSA) and cell cycle-nonspecific agents (CCNSA). Immunotherapy of tumors has two purposes: to target tumor cells and to activate immune cells. The combined use of radiotherapy and chemotherapy may enhance efficacy.

Radiotherapy and chemotherapy, like traditional cancer treatments, have disadvantages, such as nonspecific targeting, killing normal cells (including immune cells), and producing toxic side effects. Recently, chemotherapy and radiotherapy have induced the immune response by promoting the death of immunogenic tumor cells or destroying the TME. Therefore, a proper combination of immunotherapy and chemotherapy/radiotherapy can promote the reconstruction of antitumor immunity, reduce side effects, and, thus, benefit patients (Figure 1) [3].

## 2. Current Status of Cancer Immunotherapy

Immunotherapy for cancer has two purposes: to target tumor cells, such as bacterial toxins and oncolytic viruses, and to activate immune cells via cell therapy, tumor vaccines, immunological adjuvants, and immune checkpoint inhibitors [4].

Bacteria can target a hypoxic, low-pH, high-permeability TME, depending on their natural characteristics. Bacterial toxins promote tumor cell apoptosis [5]. For example, the multifunctional-autoprocessing repeat-in-toxin protein of Vibrio vulnificus can inhibit the growth of tumor cells by cutting the Ras protein [6]. Bacillus Calmette–Guérin (BCG) kills tumor cells by enhancing macrophage activity [7]. Researchers have recently used genetic engineering technology to modify bacteria to make them more targeted and penetrating.

The principle of an oncolytic virus is to selectively infect cancer cells with natural or recombinant viruses and to split cancer cells through replication of the virus itself. Meanwhile, tumor antigens released by cancer cells after splitting can cause an immune response and guide the immune system to attack other cancer cells or even remote lesions [8]. According to whether they have been modified, they can be divided into two categories: (1) viruses that preferentially proliferate in tumor cells after gene recombination, mainly including herpes simplex virus, adenovirus, and measles virus, and (2) wild-type virus strains and natural weak virus strains (such as reovirus, Newcastle disease virus, and parapoxvirus). Talimogene laherparepvec, engineered from HSV-1, has been approved by the FDA to treat melanoma [9].

Cellular immunotherapy is also known as adoptive cell therapy. The researchers extracted human autoimmune cells, cultured them in vitro to proliferate or increase the targeted killing function, and then transfused them back into the patient's body to kill cancer cells in the blood and tissues. Chimeric antigen receptor-engineered T cells (CAR-T) and TCR-T are adoptive cell therapies that modify T cells. CAR-T refers to the modified T cells that knock out the original T cell receptor (TCR) and replace it with the chimeric antigen receptor, while TCR-T refers to T cell receptor-engineered T cells. CAR-T has received the most attention due to its extraordinary capabilities in treating blood cancers such as B cell lymphoma [10] and leukemia [11]. However, due to the limited range of antigen selection, CAR-T does not perform well in solid tumors. TCR-T has a better antitumor effect against solid tumors but has major histocompatibility complex (MHC) molecular dependency. Moreover, natural killer cell therapy and tumor-infiltrating lymphocyte therapy belong to adoptive cell therapy.

Tumor vaccines use tumor antigens to induce specific cellular and humoral immunity, enhance the antitumor ability, and inhibit tumor growth [12]. According to the source, they can be divided into DNA vaccines, mRNA vaccines, long peptide vaccines, DC vaccines, viral vector vaccines, and bacterial vector vaccines. The therapeutic tumor vaccine Provenge (sipuleucel-T), the first FDA-approved cancer vaccine to treat metastatic prostate cancer, has been found to prolong the median survival of patients by 4.1 months, according to clinical studies [13]. Although most clinical trials of tumor vaccines have failed in the past decade, the development of neoantigen detection and prediction technology may add new vitality to tumor vaccine research. Chen et al. integrated the plasma membrane of Escherichia coli cells and the autologous tumor cell membrane into nanoparticles to develop a new personalized cancer vaccine. The experimental results revealed that these hybrid membrane nanoparticles induced a strong tumor-specific immune response after surgical resection of tumors and improved the survival rate of mice [14].

Immunological adjuvants can improve the immunogenicity of tumor antigens and activate an adaptive immune response, mainly including an aluminum adjuvant, pattern recognition receptor agonists, polymer materials, and peptides. Previous immunoadjuvants were limited by their poor absorption capacity and biocompatibility. Current advances in technologies such as nanotechnology have the potential to improve their efficacy [15]. Zhang et al. constructed an immunomodulatory adjuvant based on Zn-doped layered double hydroxides (Zn LDH). Peritumoral injection of Zn LDH can continuously neutralize an acidic TME and release a large amount of Zn, thereby promoting an inflammatory network composed of cytotoxic T cells, M1 tumor-associated macrophages, and natural killer cells. Furthermore, Zn LDH destroys endo-/lysosomes to prevent autophagy and induce mitochondrial damage. The released Zn activates the cGas-STING signal pathway to induce immunogenic cell death [16].

The immune checkpoint receptor on the surface of cancer cells can combine with the immune checkpoint on T cells, causing T cells to mistake them for normal cells. Checkpoint inhibitors reactivate the immune system by removing the inhibition of T cells [17]. Cytotoxic T lymphocyte-associated antigen-4 (CTLA-4) inhibitors, programmed cell death protein (PD-1) inhibitors, and programmed cell death ligand 1 (PD-L1) inhibitors have been used in the treatment of many tumors.

Although the aforementioned immunotherapies have been proven to be effective in preclinical and clinical research, their benefits are limited in most cases. This is because the persistent response is closely related to the immune status of the host. In addition, immunotherapy can cause immune-related adverse reactions [18], and some serious adverse reactions can be fatal [19]. Immunotherapy combined with radiotherapy and/or chemotherapy has become a new trend.

## 3. Immune Effects of Radiotherapy

Using high-energy radiation, radiotherapy can induce DNA damage and endoplasmic reticulum stress to kill tumor cells by releasing reactive oxygen species [20]. In the past, radiotherapy was believed to cause bone marrow suppression, reduce peripheral blood cells, and inhibit the immune system. Later research has found that radiotherapy can not only reduce the volume of the lesion in the area of the drug but also affect distant metastatic lesions, which is called the “abscopal effect.” The possible mechanism is that during radiotherapy, tumor cells release large numbers of antigens, which activate T lymphocytes after being presented by antigen-presenting cells, and then, the activated T cells act on primary and metastatic tumor cells [21].

Local radiotherapy can increase the apoptosis and necrosis of tumor cells, leading to the exposure and release of tumor-associated antigens (TAA) and thus stimulating the antitumor immune response [22]. Radiation-induced damage to DNA allows cancer cells to produce new antigens, which can also trigger immune responses [23]. In addition, to avoid being recognized by killer T cells, cancer cells reduce the expression of MHC and increase the expression of immunosuppressive surface proteins or cytokines. Radiotherapy can increase the expression of MHC molecules, stress ligands, and death receptors on the surface of tumor cells to upregulate the killing sensitivity of T cells and natural killer cells [24, 25]. However, radiotherapy also activates tumor cells to release various immunosuppressive factors, such as TGF-*β*, and recruits immunosuppressive cells, such as myeloid suppressor cells, to jointly suppress the antitumor immune response [26].

In the TME, radiotherapy activates inflammation, circulatory hypoxia, immunoregulation, revascularization, cancer-associated fibroblast- (CAF-) coordinated extracellular matrix remodeling, and fibrosis. These changes take place in an interrelated way [27]. They can increase the infiltration of immune cells into the tumor parenchyma and play a therapeutic role. For example, radiotherapy induces the expression of CXC chemokine ligands 10 and 16, which promotes T cell recruitment into the TME and increases CD8+ T cell infiltration [28].

## 4. Radiotherapy Combined with Immunotherapy

### 4.1. Radiotherapy plus Tumor Vaccines

**Figure 2:**
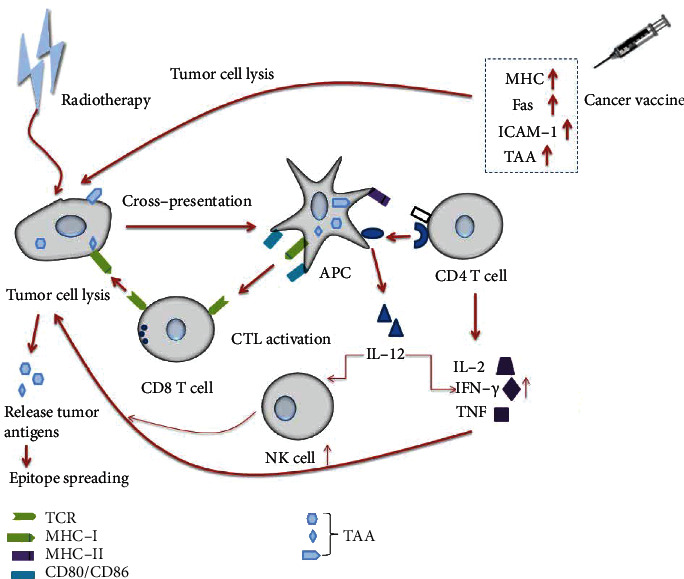
Mechanism of vaccines plus radiotherapy. CD8 T cells can recognize the tumor antigen peptide–MHCI molecule complex on the surface of tumor cells and then proliferate and differentiate into cytotoxic T lymphocytes (CTL) with a specific killing activity following activation, which mediates the necrosis or apoptosis of tumors. Exfoliating from the surface of tumor cells, tumor antigens are absorbed and processed by antigen-presenting cells (APC) as polypeptide molecules and then expressed on the surface of APC in the form of a tumor antigen peptide–MHCII molecule complex. Tumor antigen-specific CD4 T cells recognize and activate the complex, release cytokines such as interleukin-2 (IL-2) and *γ*-interferon (IFN-*γ*) to activate monocytes-macrophages and NK cells, and enhance the killing function of CD8 CTL. IFN, tumor necrosis factor (TNF), and CD4 CTL can directly kill tumor cells. The combination of vaccines and radiotherapy may produce a synergistic effect, which can enhance vaccine-mediated tumor cell lysis while upregulating MHC, Fas, intercellular cell adhesion molecule-1 (ICAM-1), and TAA [30, 31].

Witek et al. combined tumor adenoviral-mediated vaccination with radiotherapy to treat mice with colon cancer. They found that when mice were treated with a single method, tumors were only slightly reduced, but when radiotherapy was first performed, the number of antitumor immune cells increased by a factor of six. The treatment with the vaccine after one week of radiotherapy achieved the best effect [29]. Previous studies indicated that the combination of vaccines and radiotherapy enhanced the destruction of tumor cells by upregulating MHC, Fas (CD95), intercellular cell adhesion molecule-1, and TAA, thus enhancing vaccine-mediated tumor lysis in mouse models (Figure 2) [30, 31].

### 4.2. Radiotherapy plus Oncolytic Viruses

Reovirus is a wild-type oncolytic virus that can naturally target tumor cells overexpressed by the EGFR. By activating the Ras signaling pathway, the EGFR produces a phospholipase that antagonizes protein kinase R, which is dependent on double-stranded RNA, thereby promoting the replication of the oncolytic virus [32]. Radiation can further activate the Ras pathway without affecting viral replication in tumor cells [33]. McEntee et al. explored the interaction between radiotherapy and reovirus type 3 Dearing (RT3D) in melanoma cell lines with a BRAF mutant, Ras mutant, or BRAF/Ras wild-type genotype. The results depicted that RT3D combined with radiation could significantly potentiate cytotoxicity in vivo and in vitro, regardless of the genotype. The enhancement of cytotoxicity depended on the increase in viral replication, which was mediated by the upregulation of cancer upregulated gene 2 (CUG2) and the subsequent downregulation of pPKR and p-eIF2*α*, thereby activating mitochondrial apoptosis signals and promoting cell death [34].

### 4.3. Radiotherapy plus Immunoadjuvants

Zhang et al. evaluated the combination of radiotherapy and the synthetic agonist of toll-like receptor 9 (TLR9) in a mouse lung cancer model. They found that the combined therapy could induce specific IgG autoantibodies and stimulate the proliferation and activation of plasmacytoid DCs, killer DCs, and TLR9-expressing B cells. Local control of radiotherapy was improved, and systemic lung metastasis was reduced [35]. Researchers also found that systemic administration of TLR9 agonists prevented mucositis and intestinal injury induced by radiation without radiation protection for abdominal tumors [36]. Baird et al. reported that the inflammatory pathway activated by the STING ligand produced potent adjuvant activity, which enhanced the adaptive immune response to tumor antigens released by radiation. In a murine model of pancreatic cancer, they found that CT-guided radiotherapy combined with new murine and human STING ligands synergistically controlled local and distant lesions. Subsequent studies suggested that TNF*α*-dependent and T cell-independent hemorrhagic necrosis occurred in the early stages, followed by CD8 T cell-dependent control of residual lesions [37].

### 4.4. Radiotherapy plus Checkpoint Inhibitors

A clinical study observed the efficacy of local radiotherapy combined with CTLA-4 inhibitors in 50 castration-resistant metastatic prostate cancer patients. The results demonstrated that complete response (CR) was achieved in one patient, prostate-specific antigens were decreased in eight patients, and the disease remained stable in six patients [38]. Twyman-Saint et al. found that in clinical melanoma trials, patients with high programmed death-ligand 1 (PD-L1) did not respond to anti-CTLA4 plus radiation and depicted persistent T cell failure, but the combination of anti-CTLA4, anti-PD-L1, and radiation could promote immune responses. Anti-CTLA4 promotes T cell proliferation, while radiotherapy forms the TCR sequences of amplified peripheral clones. Adding a PD-L1 blocker could reverse T cell failure, attenuate the inhibition of the CD8/Treg ratio, and facilitate the expansion of oligo-clonal T cells [39]. Radiotherapy was reported to upregulate PD-L1 expression by increasing type I interferon (IFN) in the TME to avoid effector T cells [40, 41]. In addition, radiotherapy combined with a programmed cell death protein 1 inhibitor achieved surprisingly encouraging results in colorectal cancer [42], lung cancer [43], Merkel cell carcinoma [44], and other cancers. Nevertheless, some side effects (such as noninfectious inflammation) were also observed [45].

### 4.5. Radiotherapy plus Cellular Immunotherapy

In a pancreatic cancer model with heterologous expression of sialyl Lewis-a (sLeA), DeSelm et al. found that both sLeA + and sLeA- tumor cells exposed to low doses of radiation were sensitive to CAR-T, which reduced the recurrence of antigen-negative tumors. When combined with sLeA + tumor cells, CAR-T cells targeted by sLeA produced TRAIL and killed sLeA- tumor cells that had been previously exposed to radiation [46]. Xiao et al. reported a case of radiotherapy combined with CAR-T targeting B cell maturation antigens to treat refractory myeloma, in which T cell diversity changed after treatment, with more than 30% of newly expanded TCRs. A meta-analysis of 16 randomized controlled studies evaluated the efficacy and safety of cytokine-induced killer (CIK) therapy combined with radiation therapy for lung cancer. The results indicated that CIK therapy plus radiotherapy could improve the clinical response, OS, and progression-free survival (PFS). However, the combined therapy might have a high risk of fever and a low risk of leukocytopenia [47]. Radiotherapy can promote the death of tumor cells and release tumor-specific antigens [22], directly destroy the DNA of tumor cells, and generate new antigens [48]. In addition, radiotherapy can upregulate the expression of tumor MHC-I, which can better present tumor-specific antigens and enhance the visibility of tumors to immune cells.

## 5. Immune Effects of Chemotherapy

Chemotherapy drugs play an antitumor role by targeting the biological events necessary for rapid cell division. Many immune cells have the property of rapid proliferation; therefore, they may also be the target of chemotherapy. The numbers of T cells and B cells decreased significantly after receiving some chemotherapy drugs [49, 50]. Immunosuppressive cells can proliferate rapidly, hence also being sensitive to chemotherapy drugs. Gemcitabine can inhibit the activity of myeloid-derived suppressor cells (MDSC) and upregulate human leukocyte antigens on the surface of tumor cells, thereby promoting the killing effect of cytotoxic T cells [51]. Therefore, chemotherapy can be combined with CIK cellular immunotherapy with MDSC accumulation as a limitation [52].

In addition, chemotherapy-induced tumor cell death can increase the release of TAA [53]. Some chemotherapeutic agents can induce immunogenic cell death. The resulting autophagy of tumor cells releases three signals: (1) endoplasmic reticulum stress leads to calreticulin exposure. Calreticulin binds to CD91 and stimulates DC to engulf tumor antigens [54]; (2) chemotherapy drugs induce the autophagy of tumor cells by activating TLR3 on the surface of cells, and adenosine triphosphate is released to recruit DC into tumor focus; and (3) high-mobility group protein B1 is released and combined with TLR4 to activate the signal pathway [55]. The formyl peptide receptor 1 on the DC surface forms stable binding with annexin A1 on the tumor cell surface to mature the DC. The DC carries out antigen uptake and presents it to T cells [56].

Some chemotherapy drugs can increase MHC I on the surface of tumor cells and enhance tumor antigen presentation [57]. Chemotherapeutics were also reported to upregulate MHC I expression in DCs [58]. Moreover, various chemotherapy drugs can upregulate immune checkpoint ligands on the surface of tumor cells. For instance, paclitaxel can induce the upregulation of PD-L1 in ovarian cancer cells by activating the NF-*κ*B signaling pathway [59]. In bone marrow stromal cells, chemotherapy drugs can upregulate granulocyte-macrophage colony-stimulating factor and stimulate the extracellular signal-regulated kinase 1/2 signaling pathway to induce PD-L1 expression [60].

## 6. Chemotherapy Combined with Immunotherapy

### 6.1. Chemotherapy plus Tumor Vaccines

TAA mucinous glycoprotein-1 (MUC1) is abnormally expressed in various epithelial-derived tumors. The resistance of paclitaxel (PTX) to NSCLC is related to the expression of MUC1 on the surface of tumor cells. Compared with NSCLC A549 cells, PTX-resistant A549 cells (A549/PTX) had an enhanced spheroidizing ability, and MUC1-C, PI3K/P-Akt, and *β*-catenin increased significantly. Silencing MUC1 could reduce the expression of PI3K/p-Akt in tumor cells (A549/PTX) and inhibit the ability of stem cells to form spheroids [61]. Some MUC1 vaccines, such as TG4010 and L-BLP25, have demonstrated activity in clinical trials. A multicenter, unblinded, randomized phase IIB clinical study enrolled 148 patients with stage IIIB/IV NSCLC expressing MUC1. The patients received six cycles of cisplatin/gemcitabine chemotherapy with or without TG4010. The results suggested that the 6-month PFS of the combined therapy group was 43.2%, which was 35.1% in the chemotherapy group [62].

Ajani et al. conducted a multicenter phase II clinical trial in which advanced gastric cancer patients were treated with a combination of the G17DT vaccine and “platinum plus 5-fluorouracil” chemotherapy. For those patients who had immune responses after vaccination, the median survival and the time to progression were prolonged in comparison to unvaccinated patients [63].

### 6.2. Chemotherapy plus Oncolytic Viruses

Oncolytic viruses combined with chemotherapy can reduce the dosage of drugs or shorten the course of treatment under the same conditions, as well as reduce the probability of drug resistance [64]. Soliman et al. reported the safety and efficacy of neoadjuvant chemotherapy combined with talimogene laherparepvec in phase I trial of stage II or III triple-negative breast cancer. The rate of pathological complete remission of the combined therapy significantly increased. Preliminary evidence of strong immune activation, such as a significant increase in CD45R0 + T cells, was observed, and this result was further investigated in phase II trials [65]. Pelareorep (a proprietary isolate of RT3D) and gemcitabine were used to treat advanced pancreatic cancer. Among the 34 patients enrolled, 1 experienced partial remission, 23 had stable disease, and 5 experienced disease progression. The median OS was 10.2 months. The 1- and 2-year survival rates were 45% and 24%, respectively [66].

### 6.3. Chemotherapy plus Immunoadjuvants

The GOLFIG regimen, i.e., gemcitabine, oxaliplatin, levofolinate, and fluorouracil combined with recombinant IL-2 and granulocyte-macrophage colony-stimulating factor, was used to treat metastatic colorectal cancer. Caraglia et al. enrolled 179 patients for a multicenter retrospective study and reported the results of a 15-year follow-up. The mean PFS and OS were 15.28 and 24.6 months, respectively, and 14 patients did not progress within ten years [67].

### 6.4. Chemotherapy plus Checkpoint Inhibitors

Chemotherapy enhances the tumor-killing ability of immune checkpoint inhibitors by enhancing T cell activation and cancer cell infiltration [68]. A randomized controlled trial of 542 subjects showed that pembrolizumab, a humanized anti-PD1 monoclonal antibody, might improve OS in advanced urothelial carcinoma patients during or after platinum-containing chemotherapy in comparison with the chemotherapy group (12-month follow-up 59% death vs. 70% death) [69]. Rosenberg et al. assessed the efficacy of atezolizumab, which could selectively bind to PD-L1, in treating locally advanced and metastatic urothelial carcinoma after platinum chemotherapy. Atezolizumab exhibited sustained activity and good tolerance [70]. Hersh et al. compared dacarbazine combined with a CTLA-4 inhibitor (ipilimumab) and ipilimumab alone in treating patients with metastatic melanoma. The objective response rate of patients receiving dacarbazine plus ipilimumab was significantly higher than that of ipilimumab (14.3% vs. 5.4%) [71]. Phase III clinical studies revealed that the OS of the combined therapy was longer than that of dacarbazine monotherapy [72].

### 6.5. Chemotherapy plus Cellular Immunotherapy

Zhao et al. performed a retrospective study of patients with stage II–III gastric cancer after gastrectomy. A total of 53 patients received autologous CIK cells plus chemotherapy, and 112 received chemotherapy alone. Compared with the monotherapy group, the 5-year OS rate and the 5-year PFS rate of the combined therapy group significantly improved, without serious side effects observed [73]. Junghans et al. used fludarabine in combination with a cyclophosphamide regimen to shape the therapeutic microenvironment for CAR-T cell therapy. Six patients with refractory advanced prostate cancer who failed in chemotherapy and castration were treated with PSMA-targeted CAR-T combined with IL-2. The results demonstrated that the tumors were significantly reduced in two patients, and one of them had a 70% decrease in prostate-specific antigens [74].

## 7. Concluding Remarks and Future Perspectives

Cancer immunotherapy, such as CPI and CAR-T, has achieved encouraging success. Due to the low response and the acquired resistance, however, patients who can benefit are few, and some may suffer from serious side effects. An increasing number of studies have demonstrated that immunotherapy combined with radiotherapy/chemotherapy can enhance tumor immunogenicity, thereby turning “cold tumors” into “hot tumors,” with higher efficacy and fewer side effects. In addition, several studies addressing the combination of immunotherapy, chemotherapy, and radiotherapy have reported good results.

Nonetheless, there are still many issues to solve; the first of which is to choose the right patients because different clinical stages and histological types can affect clinical outcomes [75]. Second, as for combining immunotherapy with radiotherapy/chemotherapy, it is important to determine the optimal dose, timing, and sequence, which requires a deeper understanding of the underlying mechanisms. Third, the difference in patients' sensitivity to treatment should be considered, and the efficacy should be tested using specific strategies. Traditional solid tumor evaluation criteria may underestimate the therapeutic effect of immunotherapy drugs in patients with malignant tumors. Therefore, the benefits should be evaluated in conjunction with immune-related responses. Lastly, regarding radiotherapy, it is necessary to explore the best location, segmentation mode, and total dose of radiotherapy, as the degree of immune responses caused by radiotherapy varies in different organs, and conventional segmented radiotherapy and large segmented radiotherapy can also lead to different antitumor immune responses.

Only through an effective and proper combination of therapies can the tumor burden be minimized, tumor cell-induced immune suppression is relieved, and patients' immune function is improved or reconstructed, which requires more research and persistent effort. We believe that achievements in this field will bring hope to the individualized, comprehensive treatment of tumors.

## References

[1] Van Parijs L., Abbas A. K. (1998). Homeostasis and self-tolerance in the immune system: turning lymphocytes off. *Science*.

[2] Chen D. S., Mellman I. (2013). Oncology meets immunology: the cancer-immunity cycle. *Immunity*.

[3] Zitvogel L., Galluzzi L., Smyth M. J., Kroemer G. (2013). Mechanism of action of conventional and targeted anticancer therapies: reinstating immunosurveillance. *Immunity*.

[4] Sathyanarayanan V., Neelapu S. S. (2015). Cancer immunotherapy: strategies for personalization and combinatorial approaches. *Molecular Oncology*.

[5] Jiang S.-N., Phan T. X., Nam T.-K. (2010). Inhibition of tumor growth and metastasis by a combination of Escherichia coli–mediated cytolytic therapy and radiotherapy. *Molecular Therapy*.

[6] Antic I., Biancucci M., Zhu Y., Gius D. R., Satchell K. J. F. (2015). Site-specific processing of Ras and Rap1 Switch I by a MARTX toxin effector domain. *Nature Communications*.

[7] Lamm D. L., Thor D. E., Harris S. C., Reyna J. A., Stogdill V. D., Radwin H. M. (1980). Bacillus Calmette-Guerin immunotherapy of superficial bladder cancer. *Journal of Urology*.

[8] Kaufman H. L., Kohlhapp F. J., Zloza A. (2015). Oncolytic viruses: a new class of immunotherapy drugs. *Nature Reviews Drug Discovery*.

[9] Haitz K., Khosravi H., Lin J. Y., Menge T., Nambudiri V. E. (2020). Review of talimogene laherparepvec: A first-in-class oncolytic viral treatment of advanced melanoma. *Journal of the American Academy of Dermatology*.

[10] Abramson J. S., McGree B., Noyes S. (2017). Anti-CD19 CAR T cells in CNS diffuse large-B-cell lymphoma. *The New England Journal of Medicine*.

[11] Park J. H., Rivière I., Gonen M. (2018). Long-term follow-up of CD19 CAR therapy in acute lymphoblastic leukemia. *The New England Journal of Medicine*.

[12] Mellstedt H., Vansteenkiste J., Thatcher N. (2011). Vaccines for the treatment of non-small cell lung cancer: investigational approaches and clinical experience. *Lung Cancer*.

[13] Kantoff P. W., Higano C. S., Shore N. D. (2010). Sipuleucel-T immunotherapy for castration-resistant prostate cancer. *The New England Journal of Medicine*.

[14] Chen L., Qin H., Zhao R. (2021). Bacterial cytoplasmic membranes synergistically enhance the antitumor activity of autologous cancer vaccines. *Science Translational Medicine*.

[15] Ding B., Shao S., Yu C. (2018). Large-pore mesoporous-silica-coated upconversion nanoparticles as multifunctional immunoadjuvants with ultrahigh photosensitizer and antigen loading efficiency for improved cancer photodynamic immunotherapy. *Advanced Materials*.

[16] Zhang L., Zhao J., Hu X. (2022). A peritumorally injected immunomodulating adjuvant elicits robust and safe metalloimmunotherapy against solid tumors. *Advanced Materials*.

[17] Schreiber R. D., Old L. J., Smyth M. J. J. S. (2011). Cancer immunoediting: integrating immunity’s roles in cancer suppression and promotion. *Science*.

[18] Kennedy L. B., Salama A. K. S. (2020). A review of cancer immunotherapy toxicity. *CA: a Cancer Journal for Clinicians*.

[19] Lyon A. R., Yousaf N., Battisti N. M. L., Moslehi J., Larkin J. (2018). Immune checkpoint inhibitors and cardiovascular toxicity. *The Lancet Oncology*.

[20] Golden E. B., Pellicciotta I., Demaria S., Barcellos-Hoff M. H., Formenti S. C. (2012). The convergence of radiation and immunogenic cell death signaling pathways. *Frontiers in Oncology*.

[21] Postow M. A., Callahan M. K., Barker C. A. (2012). Immunologic correlates of the abscopal effect in a patient with melanoma. *The New England Journal of Medicine*.

[22] Kotera Y., Shimizu K., Mulé J. J. (2001). Comparative analysis of necrotic and apoptotic tumor cells as a source of antigen(s) in dendritic cell-based immunization. *Cancer Research*.

[23] Germano G., Lamba S., Rospo G. (2017). Inactivation of DNA repair triggers neoantigen generation and impairs tumour growth. *Nature*.

[24] Reits E. A., Hodge J. W., Herberts C. A. (2006). Radiation modulates the peptide repertoire, enhances MHC class I expression, and induces successful antitumor immunotherapy. *The Journal of Experimental Medicine*.

[25] Kwilas A. R., Donahue R. N., Bernstein M. B., Hodge J. W. (2012). In the field: exploiting the untapped potential of immunogenic modulation by radiation in combination with immunotherapy for the treatment of cancer. *Frontiers in Oncology*.

[26] Vanpouille-Box C., Diamond J. M., Pilones K. A. (2015). TGF*β* is a master regulator of radiation therapy-induced antitumor immunity. *Cancer Research*.

[27] Barker H. E., Paget J. T., Khan A. A., Harrington K. J. (2015). The tumour microenvironment after radiotherapy: mechanisms of resistance and recurrence. *Nature Reviews Cancer*.

[28] Matsumura S., Wang B., Kawashima N. (2008). Radiation-induced CXCL16 release by breast cancer cells attracts effector T cells. *Journal of Immunology*.

[29] Witek M., Blomain E. S., Magee M. S., Xiang B., Waldman S. A., Snook A. E. (2014). Tumor radiation therapy creates therapeutic vaccine responses to the colorectal cancer antigen GUCY2C. *International Journal of Radiation Oncology • Biology • Physics*.

[30] Chakraborty M., Abrams S., Camphausen K. (2003). Irradiation of tumor cells up-regulates Fas and enhances CTL lytic activity and CTL adoptive immunotherapy. *The Journal of Immunology*.

[31] Chakraborty M., Abrams S., Coleman C., Camphausen K., Schlom J., Hodge J. W. (2004). External beam radiation of tumors alters phenotype of tumor cells to render them susceptible to vaccine-mediated T-cell killing. *Cancer Research*.

[32] Shmulevitz M., Marcato P., Lee P. W. (2005). Unshackling the links between reovirus oncolysis, Ras signaling, translational control and cancer. *Oncogene*.

[33] Twigger K., Vidal L., White C. L. (2008). Enhanced in vitro and in vivo cytotoxicity of combined reovirus and radiotherapy. *Clinical Cancer Research*.

[34] McEntee G., Kyula J. N., Mansfield D. (2016). Enhanced cytotoxicity of reovirus and radiotherapy in melanoma cells is mediated through increased viral replication and mitochondrial apoptotic signalling. *Oncotarget*.

[35] Zhang H., Liu L., Yu D. (2012). An in situ autologous tumor vaccination with combined radiation therapy and TLR9 agonist therapy. *PLoS One*.

[36] Saha S., Bhanja P., Liu L. (2012). TLR9 agonist protects mice from radiation-induced gastrointestinal syndrome. *PLoS One*.

[37] Baird J. R., Friedman D., Cottam B. (2016). Radiotherapy combined with novel STING-targeting oligonucleotides results in regression of established tumors. *Cancer Research*.

[38] Slovin S. F., Higano C. S., Hamid O. (2013). Ipilimumab alone or in combination with radiotherapy in metastatic castration- resistant prostate cancer: results from an open-label, multicenter phase I/II study. *Annals of Oncology*.

[39] Twyman-Saint Victor C., Rech A. J., Maity A. (2015). Radiation and dual checkpoint blockade activate non-redundant immune mechanisms in cancer. *Nature*.

[40] Deng L., Liang H., Burnette B., Weicheslbaum R. R., Fu Y. X. (2014). Radiation and anti-PD-L1 antibody combinatorial therapy induces T cell-mediated depletion of myeloid- derived suppressor cells and tumor regression. *Oncoimmunology*.

[41] Deng L., Liang H., Xu M. (2014). STING-dependent cytosolic DNA sensing promotes radiation-induced type I interferon-dependent antitumor immunity in immunogenic tumors. *Immunity*.

[42] Seyedin S. N., Hasibuzzaman M. M., Pham V. (2020). Combination therapy with radiation and PARP inhibition enhances responsiveness to anti-PD-1 therapy in colorectal tumor models. *International Journal of Radiation Oncology • Biology • Physics*.

[43] Shaverdian N., Lisberg A. E., Bornazyan K. (2017). Previous radiotherapy and the clinical activity and toxicity of pembrolizumab in the treatment of non-small-cell lung cancer: a secondary analysis of the KEYNOTE-001 phase 1 trial. *The Lancet Oncology*.

[44] Xu M. J., Wu S., Daud A. I., Yu S. S., Yom S. S. (2018). In-field and abscopal response after short-course radiation therapy in patients with metastatic Merkel cell carcinoma progressing on PD-1 checkpoint blockade: a case series. *Journal for Immunotherapy of Cancer*.

[45] Tamiya A., Tamiya M., Nakahama K. (2017). Correlation of radiation pneumonitis history before nivolumab with onset of interstitial lung disease and progression-free survival of patients with pre-treated advanced non-small cell lung cancer. *Anticancer Research*.

[46] DeSelm C., Palomba M. L., Yahalom J. (2018). Low-dose radiation conditioning enables CAR T cells to mitigate antigen escape. *Molecular Therapy*.

[47] Xiao Z., Wang C. Q., Zhou M. H. (2018). Clinical efficacy and safety of CIK plus radiotherapy for lung cancer: a meta- analysis of 16 randomized controlled trials. *International Immunopharmacology*.

[48] Lussier D. M., Alspach E., Ward J. P. (2021). Radiation-induced neoantigens broaden the immunotherapeutic window of cancers with low mutational loads. *Proceedings of the National Academy of Sciences*.

[49] Harris J., Sengar D., Stewart T., Hyslop D. (1976). The effect of immunosuppressive chemotherapy on immune function in patients with malignant disease. *Cancer*.

[50] Sung L., Nathan P. C., Lange B., Beyene J., Buchanan G. R. (2004). Prophylactic granulocyte colony-stimulating factor and granulocyte-macrophage colony-stimulating factor decrease febrile neutropenia after chemotherapy in children with cancer: a meta-analysis of randomized controlled trials. *Journal of Clinical Oncology*.

[51] Le H. K., Graham L., Cha E., Morales J. K., Manjili M. H., Bear H. D. (2009). Gemcitabine directly inhibits myeloid derived suppressor cells in BALB/c mice bearing 4T1 mammary carcinoma and augments expansion of T cells from tumor- bearing mice. *International Immunopharmacology*.

[52] Wang Z., Liu Y., Zhang Y., Shang Y., Gao Q. (2016). MDSC-decreasing chemotherapy increases the efficacy of cytokine-induced killer cell immunotherapy in metastatic renal cell carcinoma and pancreatic cancer. *Oncotarget*.

[53] Nowak A. K., Lake R. A., Marzo A. L. (2003). Induction of tumor cell apoptosis in vivo increases tumor antigen cross-presentation, cross-priming rather than cross-tolerizing host tumor-specific CD8 T cells. *Journal of Immunology*.

[54] Garg A. D., Krysko D. V., Verfaillie T. (2012). A novel pathway combining calreticulin exposure and ATP secretion in immunogenic cancer cell death. *The EMBO Journal*.

[55] Apetoh L., Ghiringhelli F., Tesniere A. (2007). Toll-like receptor 4-dependent contribution of the immune system to anticancer chemotherapy and radiotherapy. *Nature Medicine*.

[56] Vacchelli E., Ma Y., Baracco E. E. (2015). Chemotherapy-induced antitumor immunity requires formyl peptide receptor 1. *Science*.

[57] Ohtsukasa S., Okabe S., Yamashita H., Iwai T., Sugihara K. (2003). Increased expression of CEA and MHC class I in colorectal cancer cell lines exposed to chemotherapy drugs. *Journal of Cancer Research and Clinical Oncology*.

[58] Jackaman C., Majewski D., Fox S. A., Nowak A. K., Nelson D. J. (2012). Chemotherapy broadens the range of tumor antigens seen by cytotoxic CD8(+) T cells in vivo. *Cancer Immunology, Immunotherapy*.

[59] Peng J., Hamanishi J., Matsumura N. (2015). Chemotherapy induces programmed cell death-ligand 1 overexpression via the nuclear factor-*κ*B to foster an immunosuppressive tumor microenvironment in ovarian cancer. *Cancer Research*.

[60] Yang M., Liu P., Wang K. (2017). Chemotherapy induces tumor immune evasion by upregulation of programmed cell death ligand 1 expression in bone marrow stromal cells. *Molecular Oncology*.

[61] Ham S., Kwon T., Bak Y. (2016). Mucin 1-mediated chemo-resistance in lung cancer cells. *Oncogenesis*.

[62] Quoix E., Ramlau R., Westeel V. (2011). Therapeutic vaccination with TG4010 and first-line chemotherapy in advanced non-small-cell lung cancer: a controlled phase 2B trial. *The Lancet Oncology*.

[63] Ajani J. A., Hecht J. R., Ho L. (2006). An open-label, multinational, multicenter study of G17DT vaccination combined with cisplatin and 5-fluorouracil in patients with untreated, advanced gastric or gastroesophageal cancer : the GC4 study. *Cancer*.

[64] Wennier S., Liu J., McFadden G. (2012). Bugs and Drugs: Oncolytic Virotherapy in Combination with Chemotherapy. *Current Pharmaceutical Biotechnology*.

[65] Soliman H., Hogue D., Han H. (2019). Abstract CT040: A Phase I Trial of Talimogene Laherparepvec Combined with Neoadjuvant Chemotherapy for Non-metastatic Triple Negative Breast Cancer. *Cancer Research*.

[66] Mahalingam D., Goel S., Aparo S. (2018). A phase II study of pelareorep (REOLYSIN?) in combination with gemcitabine for patients with advanced pancreatic adenocarcinoma. *Cancers*.

[67] Caraglia M., Correale P., Giannicola R. (2019). GOLFIG chemo-immunotherapy in metastatic colorectal cancer patients. A critical review on a long-lasting follow-up. *Frontiers in oncology*.

[68] Pfirschke C., Engblom C., Rickelt S. (2016). Immunogenic chemotherapy sensitizes tumors to checkpoint blockade therapy. *Immunity*.

[69] Narayan V., Kahlmeyer A., Dahm P. (2018). Pembrolizumab monotherapy versus chemotherapy for treatment of advanced urothelial carcinoma with disease progression during or following platinum-containing chemotherapy. A Cochrane Rapid Review. *Cochrane Database of Systematic Reviews*.

[70] Rosenberg J. E., Hoffman-Censits J., Powles T. (2016). Atezolizumab in patients with locally advanced and metastatic urothelial carcinoma who have progressed following treatment with platinum-based chemotherapy: a single-arm, multicentre, phase 2 trial. *Lancet*.

[71] Hersh E. M., O'Day S. J., Powderly J. (2011). A phase II multicenter study of ipilimumab with or without dacarbazine in chemotherapy-naïve patients with advanced melanoma. *Investigational New Drugs*.

[72] Robert C., Thomas L., Bondarenko I. (2011). Ipilimumab plus dacarbazine for previously untreated metastatic melanoma. *The New England Journal of Medicine*.

[73] Zhao H., Fan Y., Li H. (2013). Immunotherapy with cytokine-induced killer cells as an adjuvant treatment for advanced gastric carcinoma: a retrospective study of 165 patients. *Cancer Biotherapy & Radiopharmaceuticals*.

[74] Junghans R. P., Ma Q., Rathore R. (2016). Phase I trial of anti-PSMA designer CAR-T cells in prostate cancer: possible role for interacting interleukin 2-T cell pharmacodynamics as a determinant of clinical response. *Prostate*.

[75] Van Limbergen E. J., De Ruysscher D. K., Olivo Pimentel V. (2017). Combining radiotherapy with immunotherapy: the past, the present and the future. *The British Journal of Radiology*.

